# Decreased Leukocyte Telomere Length (LTL) Is Associated with Stroke but Unlikely to Be Causative

**DOI:** 10.1371/journal.pone.0068254

**Published:** 2013-07-05

**Authors:** Xin Jiang, Ming Dong, Jinquan Cheng, Sichun Huang, Yitao He, Kefu Ma, Bingshan Tang, Yi Guo

**Affiliations:** 1 Department of Geriatrics, the Second Clinical Medical College of Jinan University, Shenzhen, Guangdong, China; 2 Medical College, Shenzhen University, Shenzhen, Guangdong, China; 3 Shenzhen Centre for Disease Control and Prevention, Shenzhen, Guangdong, China; 4 Department of Neurology, the Second Clinical Medical College of Jinan University, Shenzhen, Guangdong, China; INRCA, Italy

## Abstract

**Aims:**

Interindividual variability in telomere length is highly heritable. Leukocyte telomere length (LTL) shortening has been shown to be associated with the process of atherosclerosis. But whether the inheritance of LTL is related to stroke is still unclear. The aim of this study was to test if telomere shortening was associated with stroke and whether this association was mainly due to inheritance or acquired cardiovascular risk factors.

**Methods:**

Our study was focused on stroke in patients and their siblings. 450 subjects were recruited into this study: 150 patients with ischemic stroke as case group, 150 siblings of patients free of stroke (sibling group) and 150 healthy people as normal control. LTL was measured by real-time Polymerase Chain Reactions. The association between LTL and the cardiovascular risk factors was also determined.

**Results:**

A significant decrease of LTL was found in case group when comparing with sibling (0.92±0.77 vs 1.68±1.24, p<0.001) and normal groups (0.92±0.77 vs 1.95±1.07, p<0.001), but no significant difference was found between sibling group and healthy control (p = 0.330). Shorter telomere length was independently associated with hypertension (p = 0.029, OR = 2.189, 95%CI:1.084–4.421), recent social pressure (p = 0.001, OR = 3.121, 95%CI:1.597–6.101), age (p = 0.004, OR = 1.055, 95%CI:1.017–1.093), HDL (p = 0.022, OR = 0.227, 95%CI:0.064–0.810) and diabetes (p = 0.018, OR = 3.174, 95%CI:1.221–8.252). Additionally, shortened length of telomere (p = 0.017, OR = 3.996, 95%CI:1.283–12.774) was an independent risk biomarker for stroke among case and sibling groups.

**Conclusion:**

The present study has demonstrated that decreased LTL might be associated with ischemic stroke but unlikely to be causative.

## Introduction

Stroke is one of the leading causes of death and long-term disability world wide, [1] with conventional cardiovascular risk factors such as abnormal lipid metabolism, smoking, diabetes, and hypertension accounting for only 50–60% of disease susceptibility. [2] The underlying pathophysiology is likely to be under the influence of both genetic and environmental factors.

Telomeres are tandem repeats of DNA sequences located at the ends of eukaryotic chromosomes with the primary function being to protect the telomeric regions from recombination and degradation. Thus, telomere length shortening is a biological clock that determines cellular ageing. [3] Recent evidence has suggested the relevance of telomere biology in human disorders, including vascular disease. [3] In cross-sectional and case–control studies, shortening of telomere length has recently been associated with chronic heart failure, [4] degenerative aortic valve stenosis, [5] coronary artery diseases, [6] and premature myocardial infarction (MI). [7] Recently, some studies reported no association of relative leukocyte telomere length with risk of incident ischemic stroke. [8], [9] On the contrary, Wang DW et al. (2012) showed positive correlation of shorter telomere length and ischemic stroke and proved LTL shortening was a strong predictor of poststroke death. [10].

Humans display a large inter-individual variation in leukocyte telomere length (LTL), which is influenced by heredity, sex, race/ethnicity, paternal age at conception and environmental exposures. Some studies in twins, siblings and families, a few of which were multigenerational, have found that LTL is heritable with an estimated heritability between 0.36 and 0.84. [11] In order to find out if the inheritance of LTL was the main cause of stroke, we examined leukocyte telomere length in stroke patients and age- and sex-frequency–matched siblings and control subjects. At the same time, we assessed lifestyle and psychological factors in relation to telomere length in stroke, sibling and control groups. Additionally, the potential role of telomere length as a biomarker of stroke was assessed.

## Methods

### Study Subjects

150 patients diagnosed as stroke [12] in the Neurology Unit of Shenzhen People’s Hospital between 1st March 2009 and 31st September 2010 were recruited (aged between 33–70 y). Shenzhen is an immigration city and more than 60 percent of the population are young people. Confirmation of stroke was based on the results of full neurological examination, computed tomography (CT), or magnetic resonance imaging (MRI) according to the International Classification of Diseases (9th Revision, codes 430–438). Stroke was defined as a sudden onset of non-convulsive and focal neurological deficit persisting for >24 hours. All subjects (cases) underwent strict neurological examination, CT, or MRI within 48 hours. Patients diagnosed with other types of stroke (transient ischemic attack, subarachnoid hemorrhage, embolic brain infarction, brain tumors and cerebrovascular malformation) and/or with severe comorbidities such as pulmonary fibrosis, endocrine and metabolic disease (except diabetes mellitus), severe inflammatory diseases, autoimmune diseases, tumors, and serious chronic diseases (e.g. Hepatic cirrhosis, renal failure) were excluded from the study. Subjects with cardioembolic stroke and documented atrial fibrillation were also excluded from our study. For each case, we chose as matched control subjects siblings who fulfilled the following criteria: same sex, age within 5 years and no stroke history. Age and sex-matched healthy subjects (control subjects) were recruited from local communities (n = 150). All protocols and methods were approved by the ethics committees of the local participating hospitals. All patients gave written informed consent.

### Data Collection and Risk Factors Definition

All patients were examined by a physician trained in stroke medicine. Among all participants, information on demographic characteristics and risk factors was collected using a structured questionnaire. The lifestyle question included: estimated daily intake of fruit, vegetables, meat, tea, and weekly alcohol consumption, current smoking habit, physical activity and social pressure. Meat consumption was defined as: whether the daily consumption of meat occupied half of the total food (or above), or if three kinds of animal meat (or above) eaten weekly and if each kind of meat intake was more than 3 times. Tea drinking was defined as five days of tea drinking per week in the past six months.

Alcohol drinking was classified as non-drinking (daily alcohol intake less than 50 ml or never drink, and this habit is maintained for one year), moderate-drinking (daily alcohol intake less than 150 ml but more than 50 ml) and excessive-drinking (daily alcohol intake more than 150 ml). Physical activity was defined as sports activity once per week more than 30 min, such as running, exercise, walking and other activities. Recent social pressure mainly refers to unemployment, no income, suffering from interpersonal relationship problem and physical disease within recent 2 months. Hypertension was defined by pharmacological treatment for hypertension, systolic blood pressure≥140 mm Hg, and/or diastolic blood pressure≥90 mm Hg. Diabetes was diagnosed by a fasting glucose level of >7.8 mmol/L and/or a glucose level of >11.1 mmol/L at 2 h after oral glucose challenge. Hyperlipidemia was defined as total plasma cholesterol level of >6.50 mmol/L or plasma triglyceride >2.30 mmol/L and/or low-density lipoprotein >4.14 mmol/L. Current smoking was defined as active smoking or smoking cessation <3 months. Body mass index (kg/m^2^) (BMI) was calculated from measurements of height and weight. The cut-off of BMI for overweight was >25.

### Ultrasonic Measurements

All scans were performed using a Philips (ATL) HDI 5000 duplex scanner (Seattle, USA). All subjects were studied in supine position. Neck was fully exposed with face towards opposite side. Scanning of the common carotid artery (CCA) (proximal, middle and distal portions), the carotid bifurcation (BIF), the beginning of the internal carotid artery (ICA) and external carotid artery (ECA) were obtained along the longitudinal sternocleidomastoid muscle and then along cross sections. The intima- media thickness (IMT) complex of the far wall of the common carotid artery (IMTcc) was measured at its thickest part (mean of three readings) on both transverse and longitudinal sections. The mean of the measurements from both carotid arteries was used in the analysis. IMT ≥1.2 mm at CCA was defined as medial thickening. IMT ≥1.5 mm is defined as atherosclerotic plaque. The subjects were divided into carotid intima thickening group and carotid artery plaque group. The two ultrasonographers who performed the ultrasonic scans were blinded to each subject’s background.

### Biochemical Measurements

Fasting whole blood ethylenediamine tetraacetic acid (EDTA) was collected within 2 hours. Routine blood test, liver and kidney function, fasting glucose, blood lipids, cardiac enzymes and serum uric acid were measured using the LX4201-type automatic biochemical analyzer (BECKMAN COULTER, USA). Fasting serum total homocysteine (tHcy) was determined by enzymatic commercial kits (Carolina Liquid Chemistries, USA) with an Olympus AU640 automatic analyzer.

### Telomere Measurement

All DNA samples were amplified using 10 µL PCR. DNA was extracted from whole blood in EDTA with a commercially available method (QIAmp DNA Blood Maxi kit, Qiagen) according to the manufacturer’s instructions. Mean leukocyte telomere length was measured with a quantitative real-time PCR. The thermal cycling profiles for both amplicons began with 95°C incubation for 10 minutes, followed by 30 cycles of 15 seconds at 95°C and 1 minute at 56°C for the telomere PCR or followed with 35 cycles of 15 seconds at 95°C and 1 minute at 58°C. The specificity of all amplifications was determined by melting curve analysis. Briefly, the relative telomere length was calculated as the ratio of telomere repeats to single-copy gene (SCG) copies (T/S ratio). For each sample the quantity of telomere repeats and the quantity of SCG copies were determined in comparison to a reference sample in a telomere and a SCG quantitative PCR, respectively. The mean±SD coefficients of variation were 6.7±1.3% for T/S ratio. All measurements were performed blinded with respect to clinical data. All tests were performed on the Rotor-Gene 6000 (Corbett Research Ltd, Cambridge, UK).

### Statistical Methods

Statistical analysis was performed with SPSS 16.0.1 (SPSS Inc., Chicago, IL, USA). All values are presented as mean±S.D. A value of P<0.05 was considered statistically significant. Because the length of telomere was in a non-normal distribution, data were log transformed as appropriate. Comparisons between three groups (case, sibling and normal control) were analyzed by ANOVA. The independent t-test (for numeric variable) and Pearson’ s x^2^-test (for categorical variable) were used for two groups comparison. Among all the telomere length of the subjects (case, sibling and normal control), we chose the median level of total LTL to separate the subjects into two groups: longer telomere length group (marked as 0) and shorter telomere group (marked as 1). Statistical significant association variables were put into stepwise analysis and the dependent variable was LTL shortening.

## Results

### General Characteristics of the Study Subjects

The characteristics of the study population are shown in Table 1. Stroke patients had more diabetes, hypertension and social pressure. Around 40% of stroke patients were current smokers, higher than in sibling and normal groups. Subjects in normal group had relative better lifestyle, such as more physical exercise, less tea drinking and more daily fruit. Mean LTL was successfully determined in all 450 subjects in this study. There was a significant decrease trend of LTL among stroke patients (0.92±0.77), sibling (1.68±1.24, p<0.001) and normal (1.95±1.07, p<0.001) groups. However, there was no obvious difference between sibling and normal groups (p = 0.330). In this study, most subjects were from southern China. Only 80 subjects (40 cases and 40 siblings) were from northern China. Because sometimes the siblings always grow up in the same place, the hometown of the sibling in this study was recorded same as the case. The LTL of the northern subjects (40 cases and 40 siblings) was borderline lower than that of the subjects from the south (110 cases and 110 siblings) (1.25±0.59 vs 1.71±0.61, p = 0.051). There were 290 subjects (150 cases and 140 siblings) who had IMTcc measured and were divided into four groups, normal group (n = 122, LTL: 1.8943±1.1644), intima-media thickness group (n = 43, LTL: 1.7871±0.9618), unstable carotid plaques group (n = 111, LTL: 0.9650±0.7315) and severe carotid stenosis group (n = 34, LTL: 1.1248±0.73). No significant difference between normal and intima-media thickness groups (p = 0.815), neither between unstable carotid plaques and severe carotid stenosis groups (p = 0.684) were found. However, LTL of the first two groups was higher than that of the latter two groups (p = 0.018).

**Table 1:** Characteristics of study subjects in different groups.

	Stroke group N = 150	Sibling group N = 150	Normal control N = 150	p
Age (years)	52.4±8.8	50.3±7.9	52.2±8.1	0.403
Men (n)	111	111	111	1.000
Body overweight (n)	79	67	66	0.275
Diabetes (n)	54	30	8	<0.001
Current smoker (n)	60	47	40	0.044
Hyperlipidemia (n)	47	43	47	0.845
Hypertension (n)	77	49	10	<0.001
Physical exercise (n)	26	60	90	<0.001
Recent social pressures (n)	60	32	36	<0.001
Alcohol drinking(no/less/more)	120/15/15	126/8/16	122/6/22	0.168
Meat consumption (n)	79	73	118	<0.001
Tea drinking (n)	73	77	64	0.261
Daily fruit (n)	57	62	75	0.096
SBP(mmHg)	149.4±22	134.3±22	124±11	<0.001
DBP(mmHg)	92.5±12.35	85.2±11.21	77.0±12	<0.001
TC(mmol/l)	4.88±1.03	5.02±1.07	5.40±1.30	0.021
HDL(mmol/l)	1.05±0.35	1.04±0.36	1.18±0.33	0.001
TG(mmol/l)	1.85±1.12	1.79±1.57	1.41±0.71	0.047
LDL(mmol/l)	3.17±0.92	3.26±0.91	3.35±0.90	0.667
Fasting glucose(mmol/l)	6.7±2.8	5.9±2.0	5.3±1.1	<0.001
hs-CRP(mg/l)	5.97±6.61	3.63±4.45	1.13±1.54	<0.001
Creatinine	81.55±35.78	80.50±42.18	78±23.15	0.761
Uric acid	364.91±110.87	364.22±92.85	355±80.92	0.891
tHcy (umol/l)	13.82±5.89	14.45±10.45	11.23±4.45	0.784
LTL(T/S)	0.92±0.77	1.68±1.24	1.95±1.07	<0.001

SBP systolic blood pressure, DBP diastolic blood pressure, TC Total cholesterol, HDL high-density lipoprotein, TG Triglycerides, LDL low-density lipoprotein, LTL leukocyte telomere length.

### Effects of Life Style and Cardiovascular Risk Factors on Telomere Length

The associations of life style and cardiovascular risk factors with telomere length were studied. In this study, LTL correlated negatively with age (r = -0.272, p<0.001). The mean age of women was 49.91±11.05, and 50.02±0.91 in men. However, no significant difference of LTL was observed between women and men (1.45±0.85 VS 1.54±1.22, p>0.05). Total subjects (stroke, sibling and normal control) were divided into two groups according to the median level of LTL (T/S = 1.3164). Hypertension, diabetes, recent social pressure, tea drinking, HDL, TC, hs-CRP, SBP and DBP were significant different in these two groups (Table 2). On the contrary, no significant difference in gender, hyperlipidemia, current smoker, meat consumption, alcohol drinking, body overweight, active physical exercise and daily fruit were found between shorter LTL group and longer LTL group (Table 2). In a stepwise multivariable analysis that included smoking, tHcy and all the variables associated with LTL in the univariable analysis, and with age and sex in the model, only hypertension (p = 0.029, OR = 2.189, 95%CI:1.084–4.421), recent social pressure (p = 0.001, OR = 3.121, 95%CI:1.597–6.101), age (p = 0.004, OR = 1.055, 95%CI:1.017–1.093), HDL (p = 0.022, OR = 0.227, 95%CI:0.064–0.810) and diabetes (p = 0.018, OR = 3.174, 95%CI:1.221–8.252) remained in the model (R^2^ = 0.61 p = 0.001 for the model) (Table 3).

**Table 2:** Correlations of lifestyle and laboratory parameters with LTL.

	Shorter LTL n = 225	Longer LTL n = 225	p
Age (years)	48.36±8.67	51.71±8.88	0.006
Male (n)	165	168	0.747
Hypertension (n)	91	45	<0.001
Diabetes (n)	62	30	<0.001
Hyperlipidemia (n)	77	60	0.082
Current smoker (n)	82	65	0.087
Alcohol drinking (more)(n)	28	25	0.661
Body overweight (n)	116	96	0.059
Recent social pressures (n)	88	40	<0.001
Physical exercise (n)	80	96	0.122
Tea drinking (n)	120	94	0.014
Daily fruit (n)	93	101	0.446
Meat consumption (n)	125	145	0.054
LDL(mmol/l)	3.23±0.98	3.33±0.94	0.439
HDL(mmol/l)	1.05±0.22	1.20±0.32	<0.001
TC(mmol/l)	4.96±1.05	5.27±1.11	0.044
TG(mmol/l)	1.83±1.41	1.55±1.22	0.130
Fasting glucose (mmol/l)	6.07±2.11	5.60±1.94	0.110
hs-CRP(mg/l)	6.47±8.45	3.19±10.37	0.032
Creatinine	81.97±25.34	80.23±32.54	0.879
Uric acid	365.25±98.32	362.79±89.82	0.731
tHcy (umol/l)	14.43±9.67	11.75±12.69	0.917
SBP (mmHg)	141.19±20.85	129.59±18.21	0.013
DBP(mmHg)	87.70±12.20	81.35±12.76	0.021

**Table 3:** Best model for association between telomere length (LTL) and lifestyle and laboratory parameters in a step wise multivariable model including all significant factors from univariable analyses in all subjects.

variables	Stepwise analysis		
	p	OR	95%CI
Hypertension	0.029	2.189	1.084–4.421
Recent social pressures	0.001	3.121	1.597–6.101
Age (years)	0.004	1.055	1.017–1.093
HDL (mmol/l)	0.022	0.227	0.064–0.810
Diabetes	0.018	3.174	1.221–8.252

Dependent variable is LTL shortening (LTL of less than the mean value was marked as 1, LTL of longer than the mean value was marked as 0).

### Leukocyte Telomere Length was an Independent Risk Biomarker for Stroke

In order to find out the associations of leukocyte telomere shortening and cardiovascular risk factors for stroke, we compared the stroke and sibling groups. Age, hypertension, diabetes, body overweight, recent social pressure, active physical exercise, HDL, fasting glucose, SBP, DBP and LTL were significantly different between stroke and sibling. Finally, a stepwise multivariable analysis showed that only hypertension (p = 0.014, OR = 4.658, 95%CI:1.360–15.347), the length of telomere (p = 0.017, OR = 3.996, 95%CI:1.283–12.774), recent social pressure (p = 0.007, OR = 3.874, 95%CI:1.455–10.313) and HDL (p = 0.029, OR = 0.015, 95%CI:0.000–0.654) were independent risk factors for stroke (Table 4).

**Table 4:** Best model for association of cardiovascular risk factors and LTL to stroke in a step wise multivariable model including all significiant risk factors from univariable analyses between stroke and sibling subjects.

Variables	Stepwise analysis		
	p	OR	95%CI
Hypertension (n)	0.014	4.658	1.360–15.347
Recent social pressures (n)	0.007	3.874	1.455–10.313
HDL(mmol/l)	0.029	0.015	0.000–0.654
LTL	0.017	3.996	1.283–12.447

## Discussion

In the present study, we studied the effects of life style and cardiovascular risk factors on telomere length. We found that leukocyte telomere length was significantly shorter in stroke patients than that of the case-sibling or normal control, but no significant difference was observed between the latter two groups. The shortened telomere length might be related to the higher blood pressure, increased social pressure recently, raised lipids and diabetes medical history and age in the stroke group. In addition, we further examined that decreased LTL might be an associated factor with ischemic stroke rather than being causative.

### Cardiovascular Risk Factors with Telomere Length

Lifestyle factors are known to promote cardiovascular disease which might also adversely affect the telomere length. For example, increases in obesity and insulin resistance over 10–13 years are associated with decreases in telomere length. [13] A previous study showed that a significant difference in LTL across the geographical regions of Europe was observed. [14] In China, the stroke rate is higher in the North than in the South because of different lifestyles, environment and genetics. In this study, the LTL of the northern subjects was borderline lower than that of the subjects in southern (1.25±0.59 vs 1.71±0.61, p = 0.051). That might be due to the small sample size. Previous studies proved that smoking was associated with shorter telomeres in normal subjects [15] as well as in lean and obese women [16], while greater physical activity in leisure time has been associated with longer telomeres in healthy twins [17].In addition, a recent study has addressed that improvements in nutrition and lifestyle are associated with increased telomerase activity. [18] Subjects on a mixed diet had a tendency towards reduced telomeres length when compared with vegetarians (p = 0.088). [19] All these observations show that lifestyle plays an important role in the length of telomeres. However, proper physical activity and a healthy diet may slow down the shortening of telomere, and delaying aging.

In this study, no significant association was observed between body weight (BMI) (p = 0.059) or physical exercise (p = 0.122) and telomere length, although the number of obese people was relatively higher and sporty people was lower in shorter LTL group. This differs with the previous study. [17] It seems more likely that the effect of mild exercise on telomere length, if any, is small and therefore difficult to detect, particularly when physical activity is assessed by general questionnaires. More importantly, the target of the previous study was focused on healthy subjects. Stroke is a complication of atherosclerosis and LTL is directly affected by smoking, obesity or less physical exercise all of which are risk factors for atherosclerosis and hence stroke. We did found that the LTL was higher in normal and intima-media thickness groups when comparing with that of unstable carotid plaques and severe carotid stenosis groups (p<0.05). However, in the current study, the nature of the diet including meat and fruit intake, tea and alcohol drinking had weak effect on LTL shortening in stroke subjects.

Interestedly, we proved that ongoing recent social pressure was an independent risk factor for telomere shortening (OR = 3.121, 95% CI: 1.597–6.101). Similarly, telomere length was significantly shorter in those with mood disorders. [20] Some studies have found that psychological stress is associated with indicators of accelerated cellular and organismal aging: oxidative stress, telomere length, and telomerase activity. [21] A current study has shown that, at the cellular level, environmental stress may promote earlier onset of age-related diseases. [22] Moreover, in this study, the effect of undergoing social pressure to telomere length was higher than that of hypertension (OR: 3.121(1.597–6.101) vs 2.189(1.084–4.421)). Thus, psychological stress (including chronic and recent pressure) might play a more important role in shortening LTL than what we have assumed. In this study, the questionnaires were asked in hospital when the patients during the first admission. We tried to ask about lifestyle and stress conditions before the event but naturally after the event there maybe bias in favour of some factors deemed to be more important to the patient or family.

A previous study demonstrated that HDL cholesterol was associated with a significant variation in LTL in a very healthy and young population, suggesting that the relationship between LTL and risk factors might be an early phenomenon. [23] In our study, we found that HDL cholesterol was independently negatively associated with LTL shortening and stroke. The same correlation has been shown in a recent study where LTL was determined in the same subjects in childhood and adulthood [24] and in cross-sectional studies conducted in higher risk populations. [25] A putative explanation for this association is that HDL-cholesterol has anti-oxidant and anti-inflammatory properties and LTL ostensibly reflects the cumulative burden of inflammation and oxidative stress. [26] Low HDL-cholesterol phenotypes have been shown to display elevated oxidative stress and accelerated senescence suggesting that a reduced LTL could be a biomarker of a status of increased oxidative stress and inflammation. [27].

### Telomere Length Shortening and Stroke

It is well established that hypertensive subjects are at higher risk for atherosclerosis and an accelerated cardiovascular aging. However, not all hypertensive patients ultimately manifest cardiovascular complications. The reasons for this are unknown but may reflect environmental and genetic factors such as oxidative stress, inflammation, and other molecular and cellular mechanisms that are involved in aging. http://hyper.ahajournals.org/content/43/2/182.long - ref-2#ref-2Telomere length in white blood cells may register the cumulative burden of oxidative stress and inflammation in the circulation during an individual’s lifetime. Numerous studies proved the telomere length shortening occurs in cardiovascular and cerebral diseases. Heredity is a well recognized major component [28], [29] and for which the paternal influence seems to be of most importance. [30] Furthermore, this shortening was thought to occur before the atherosclerosis and consequent clinical events are apparent. However, few studies have focused on stroke in siblings and most of them compared with normal controls.

In this study, we chose the brothers and sisters of the patients as the comparison group. The telomere length of stroke subjects was significantly shorter than that of siblings and normal controls. However, we did not find any obvious difference of telomere length between the sibling and normal controls. It might because the telomere shortening in stroke is mainly due to the acquired risk factors such as pressure, hypertension, age, diabetes and oxidative stress although the inherited factor is also important. Overall, due to the cell division, the telomere shortening rate is gradually increased. So the leukocyte telomere shortening in patients with cardiovascular disease may partly be the result of increased aging which is induced by chronic inflammation as part of the atherosclerosis process. It has been reported that the telomere length can be affected by environmental factors and physical exercise. Satoh M. has demonstrated that oxidative treatments induced telomere shortening and decrease in telomerase activity of endothelial progenitor cells (EPS). [31] They also proved intensive lipid-lowering therapy may prevent EPS telomere erosion in patients with coronary artery disease. [32] Statin treatment may delay cell senescence and promote DNA repair, including telomere shortening, in atherosclerosis. [33] Ornish et al reported these increases in telomerase activity as a significant association rather than inferring causation. [18].

Furthermore, in order to eliminate the associations of heredity stroke subjects and sibling subjects were combined together and studied. In this combined cohort, telomere length was chosen as an independent risk biomarker of stroke. Although numerous studies proved telomere length shortening a biomarker for ongoing processes leading to cardiovascular diseases, few have focused on stroke and their relatives. Our results suggested that the lifestyle and stress could influence the blood cell telomere length attrition rate, which might play an important role in stroke pathogenesis. However, in this study, we just showed a strong association of telomere length shortening and stroke and telomere length shortening might be a good risk biomarker for stroke; as for causality, we might need further experiments to explore.

In summary, these findings suggested that leukocyte telomere length was influenced by lifestyle and psychological pressure. Decreased telomere length was independently associated with stroke but it is more likely to be the result of the disease processes rather than the cause.

### Limitations

The limitation of this study is the relative small sample size. This study is based on a very selected group of young Chinese stroke patients (mean 52 years) and the generalisability is relatively limited. It would be better if we can study the telomere length in stroke in twins. Future studies are necessary to confirm the underlying mechanisms of telomere in stroke process.
